# Development of Human Vectored Brucellosis Vaccine Formulation: Assessment of Safety and Protectiveness of Influenza Viral Vectors Expressing *Brucella* Immunodominant Proteins in Mice and Guinea Pigs

**DOI:** 10.1155/2020/1438928

**Published:** 2020-11-19

**Authors:** Dina Bugybayeva, Sholpan Ryskeldinova, Nadezhda Zinina, Makhpal Sarmykova, Nurika Assanzhanova, Zhailaubay Kydyrbayev, Kaissar Tabynov

**Affiliations:** ^1^Research Institute for Biological Safety Problems, 15 Momushuly, Gvardeyskiy 080409, Kazakhstan; ^2^Kazakh National Agrarian University, 8 Abay Avenue, Almaty 050010, Kazakhstan; ^3^Research Institute of Cardiology and Internal Medicine, 120 Aiteke bi, Almaty 050000, Kazakhstan

## Abstract

In this paper, we first used recombinant influenza viral vector (rIVV) subtype H5N1 expressing from the open reading frame of NS1 80 and NS1 124 amino acids of *Brucella* outer membrane proteins (Omp) 16 and 19, ribosomal L7/L12, and Cu-Zn superoxide dismutase (SOD) proteins to develop a human brucellosis vaccine. We made 18 combinations of IVVs in mono-, bi-, and tetravalent vaccine formulations and tested them on mice to select the safest and most effective vaccine samples. Then, the most effective vaccine candidates were further tested on guinea pigs. Safety of the rIVV-based vaccine candidate was evaluated by a mouse weight-gain test. Mice and guinea pigs were challenged with the virulent strain *B. melitensis* 16M. The protective effect of the rIVV-based vaccine candidate was assessed by quantitation of *Brucella* colonization in tissues and organs of challenged animals. All vaccine formulations were safe in mice. Tested vaccine formulations, as well as the commercial *B. melitensis* Rev.1 vaccine, have been found to protect mice from *B. melitensis* 16M infection within the range of 1.6 to 2.97 log_10_ units (*P* < 0.05). Tetravalent vaccine formulations from the position of NS1 80 amino acids (0.2 ± 0.4), as well as the commercial *B. melitensis* Rev.1 vaccine (1.2 ± 2.6), have been found to protect guinea pigs from *B. melitensis* 16M infection at a significant level (*P* < 0.05). Thus, tetravalent vaccine formulation Flu-NS1-80-Omp16+Flu-NS1-80-L7/L12+Flu-NS1-80-Omp19+Flu-NS1-80-SOD was chosen as a potential vaccine candidate for further development of an effective human vaccine against brucellosis. These results show a promising future for the development of a safe human vaccine against brucellosis based on rIVVs.

## 1. Introduction

Brucellosis is a zoonotic disease that is transmitted between species from animals to humans and is one of the most common infectious diseases in the world, with prevalence in developing countries. Annually, more than 500,000 new cases of human brucellosis are reported [1], and the main pathogenic Brucella species are *B. abortus*, *B. melitensis*, and *B. suis* [2]. Worldwide, *B. melitensi*s is the most prevalent and virulent species causing severe infections in humans [3].

Due to economic and public health consequences of brucellosis in developing countries, efforts have been made to eradicate the disease through vaccination of the livestock sector, as high incidence of the disease in the human population is largely related to the high persistence level of the brucellosis infection in livestock [4].

Various vaccine strategies have been made to develop effective brucellosis vaccines with the majority of them intended for human use [5–13]. In particular, vector-based systems on the platform of attenuated viruses (Semliki Forest virus) or bacteria (*Ochrobactrum anthropi*, *Yersinia enterocolitica*, and *Escherichia coli*) have been developed and tested as vaccine candidates against brucellosis, including the presenting recombinant of *Brucella* proteins as Cu-Zn SOD, L7/L12, and Omp19 [14–21]. These vector platforms were tested predominantly in mouse models and induced vigorous Th1-type immune responses. In the majority of studies, protection was not confirmed against a challenge with virulent bacterial strain.

*Brucella* antigens, such as outer membrane proteins (Omp) 16 and 19, ribosomal L7/L12, and Cu-Zn superoxide dismutase (SOD), are inducing a strong cell-mediated response required to clear the infection [22–24]. However, a protective effect of a single epitope of immunization versus a mixed epitope vaccination is not very well understood yet.

The *Brucella* recombinant influenza viral vector- (IVV-) based vaccine Flu-BA has been developed previously and demonstrated efficacy in bovine comparable with the commercial *B. abortus* S19 vaccine [25]. The Flu-BA was registered in Kazakhstan in 2019 for vaccination of cattle against *B. abortus* infection. Bovine species, in general, are not a very susceptible host for influenza A viruses [26]. However, the influenza viral constructs can be more effective in developing a vaccine against human brucellosis, because influenza A is a frequent infection of humans. We believe that rIVV based on subtype H5N1 has more potential as a vaccine vector due to the lack of preexisting immunity to H5N1 in the general human population [27]. Accordingly, we utilized the rIVV of the H5N1 subtype inserted with *Brucella* antigens to develop a novel vaccine candidate against human brucellosis.

Thus, the purpose of this study was to select the most optimal vaccine constructs among the safest and protective rIVV expressing *Brucella* immonodominant proteins Omp16, Omp19, L7/L12, or SOD as a potential candidate for human vaccine development.

## 2. Materials and Methods

### 2.1. Generation of Virus Constructs and Preparation of Vaccine Samples

IVVs were obtained with the standard reverse genetic method utilizing 8 bidirectional plasmids pHW2000 [28]. In this study, we used eight monovalent vaccine constructs comprising the recombinant influenza A virus of the subtype H5N1 from the A/chicken/Astana/6/05 strain expressing the *Brucella* immunodominant proteins L7/L12, Omp16, Omp19, or Cu-Zn SOD containing a sequence of 80 or 124 N-terminal amino acids from the open reading frame (ORF) of the NS1 gene (HSC Development GmbH, Austria). A detailed procedure of rIVV generation is described previously [29]. Briefly, Vero cells were cotransfected by the Lonza Nucleofector™ (Cologne, Germany) technique with plasmids encoding the PB1, PB2, PA, NP, and M genes and chimeric NS gene of the А/Puerto Rico/8/34 (H1N1) virus and the hemagglutinin (HA) and neuraminidase (NA) taken from the A/chicken/Astana/6/05 (H5N1) strain. Attenuation of the HA protein sequence of the H5 virus was provided by exchanging its polybasic cleavage site to one that included a trypsin-dependent sequence. The NS1 gene was modified to insert *Brucella* sequences. A sequence of 80 or 124 N-terminal amino acids from the NS protein paired with a sequence of *Brucella* proteins.

The obtained influenza vectors (Flu-NS1-80-Omp16, Flu-NS1-80-L7/L12, Flu-NS1-80-Omp19, Flu-NS1-80-SOD, Flu-NS1-124-Omp16, Flu-NS1-124-L7/L12, Flu-NS1-124-Omp19, and Flu-NS1-124-SOD) were then used for producing vaccine samples in 10-day-old embryonated chicken eggs (CE) as reported previously [29]. The insertion of referred *Brucella* proteins in the NS1 gene was confirmed by reverse transcription polymerase chain reaction (RT-PCR) Figure S1. Obtained allantoic fluid containing IVV inserted with various *Brucella* antigenic genes used as a mono formulation or pooled together in a 1 : 1 or 1 : 1 : 1 : 1 ratio to obtain overall 18 rIVV mono or combinations of bivalent and tetravalent formulations.

### 2.2. Bacterial Strains

The virulent strain of *B. melitensis* 16M, obtained from the Research Institute for Biological Safety Problem's (RIBSP) collection of microorganisms, was used in this study. The bacterial cells were cultured in *Brucella* base agar (Himedia Laboratories, India) under aerobic conditions at 37°C. All laboratory experiments with live *Brucella* cells were conducted in biosafety level (BSL) 3. Challenged mice and guinea pigs were kept in animal BSL 3 facility.

### 2.3. Bioethics and Animal Groups

This study was carried out in accordance with the recommendations of the national and international guidelines on animal care and use. The study protocol was approved by the IACUC of the RIBSP (approval no. 0418/04). Animals were housed in a 12 light/12 dark cycle in cages under controlled environmental conditions and were fed *ad libitum* with standard rodent diet and had no water restrictions. Experimental and control groups of animals were kept in different rooms during the entire experiment. This study used male BALB/c mice (Charles River) aged 5-7 weeks old weighing 20-25 g and conventional bred female guinea pigs weighing 300-350 g (National Center for Expertise of Drugs, Medical Products and Equipment, Kazakhstan). By the randomization method, mice were divided into 20 groups (*n* = 12 per group): 18 experimental prime-boost groups, one negative PBS (phosphate-buffered saline) control group, and one positive control group. The guinea pigs were divided into 7 groups (*n* = 5 per group): five experimental prime-boost groups, one negative (PBS) control group, and one positive control group.

### 2.4. Immunization of Mice

Groups of mice in experimental prime-boost groups (*n* = 5 or *n* = 12 per group) for safety or challenge studies were injected peritoneally (*i.p.*) twice with prepared vector brucellosis vaccine formulations (18 overall) at an interval of 14 or 21 days. After selection of mice within these 18 formulations, 5 vaccine formulations that demonstrated significant results were further tested on guinea pigs with the same prime-boost immunization scheme when rIVV was administered intranasally (*i.n.*) twice with an interval of 21 days between vaccinations. The animal immunization scheme is presented in Table 1. Mice and guinea pigs from the positive control groups were injected subcutaneously (*s.c.*) with the *B. melitensis* Rev.1 (Antigen LLP, Kazakhstan) only once at a dose of 6.0 log_10_ CFU/animal. Mice and guinea pigs of the negative control groups *s.c.* injected with 200 *μ*l of PBS.

### 2.5. Assessment of Mono-, Bi-, and Tetravalent Vaccine Formulation Safety

To assess the safety of vaccine samples (or their level of attenuation), vaccinated mouse body weight changes were monitored daily for 28 days after both prime and booster vaccinations with viral constructs or *B. melitensis* Rev.1 (vaccination was provided only once) and compared to the negative (PBS) control group. General observation for safety was evaluated by animal survival and animals' general condition, behavior, and dynamics of body weight change.

### 2.6. Assessment of the Vaccine Formulation's Protectiveness in Mice and Guinea Pigs

In order to evaluate vaccine formulation's protectiveness, mice from the experimental vaccine groups (*n* = 5 per group) and PBS injected control group (*n* = 5) were challenged *i.p.* with the virulent strain of *B. melitensis* 16M in a dose of 6.0 log_10_ CFU/animal on 21 days after the boost vaccination. Mice in the positive control group (*n* = 5) were immunized with the vaccine *B. melitensis* Rev.1 and challenged on day 42. On day 14 postchallenge, all animals were sacrificed by CO_2_ asphyxiation to collect spleen tissues aseptically for bacteriological studies. The spleen from each animal was harvested and homogenized in 5 ml of 0.1% Triton-PBS, and 100 *μ*l of 10-fold serial dilutions of spleen suspension was plated in triplicate onto *Brucella* base agar (HiMedia Laboratories, India) plates. Plates were incubated at 37°C for 2 weeks, and the number of bacterial colony growth was counted periodically by performing standard plate counts to determine the concentration of bacteria (CFU/tissue) in spleens. An animal was considered to be infected if one or more *Brucella* colonies were present in the cultures. The protective effect of the vaccine samples was assessed by comparing the degree of spleen infection of vaccinated experimental and reference control groups and unvaccinated control group after virulence challenge with *B. melitensis* 16M (expressed as log_10_ CFU/g of tissue protection unit).

After studying the protective efficacy of vaccine formulations, the five most protective vaccine formulations were assessed in the guinea pig model. The challenge study for guinea pigs was similar as what was done for mice, and it was consistent based on route of infection, grouping of animals, preparing suspension from animal organs, bacteria growing conditions, and counting of colonies. Twenty-one days after the boost vaccination, guinea pigs from the experimental groups (*n* = 25), PBS control (*n* = 5), and positive control (*n* = 5) (on day 42 after prime vaccination with vaccine *B. melitensis* Rev.1) groups were challenged *s.c.* with the virulent strain of *B. melitensis* 16M in a dose of 1.3 log_10_ CFU/animal. Thirty days after challenge, animals from all groups were euthanized and aseptically dissected to collect the following tissues: retropharyngeal, lower cervical, right and left inguinal lymph nodes, liver, spleen, and bone marrow. The results of the bacteriological assessment were evaluated from three parameters, including vaccination efficacy (expressed in %) determined as the number of animals from which no colonies were isolated, infection index (number of tissues from which *Brucella* were isolated in), and protective efficacy evaluated as the degree of *Brucella* colonization in organs and lymph nodes expressed as log_10_ CFU/g of tissue.

### 2.7. Statistical Analysis

The safety of vaccine formulations was analyzed using one-way ANOVA (Dunnett's multiple comparison test). Protection of vaccines and index of infection data were analyzed with a one-way ANOVA (Tukey's multiple comparison test) and two-way ANOVA (Sidak's multiple comparison test), respectively. The variance in protective efficacy of animal groups was compared by one-sided Fisher exact test. *P* values < 0.05 were considered significant. Means are reported with standard errors (SEM) and 95% confidence interval. Statistical analysis was performed with GraphPad Prism Software, version 6.0 (GraphPad Software Inc., La Jolla, CA, USA). The experiments have been repeated, and the results were reproducible.

## 3. Results

### 3.1. Assessment of Various Vaccine Formulations' Safety in Mice

The safety or degree of attenuation of various vaccine formulations, comprising mono-, bi-, or tetravalent rIVV, were determined in mice in comparison with the positive (*B. melitensis* 16M) and negative (PBS) control groups. It was found that all vaccine samples, including *B. melitensis* Rev.1, were safe in mice after *i.p.* injection. No animal death or disease sign was observed in any group by the end of the observation period. Overall, condition of animals both in the control and experimental groups was moderate, in terms of physical activity, appetite, and general outward condition.

**Figure 1:**
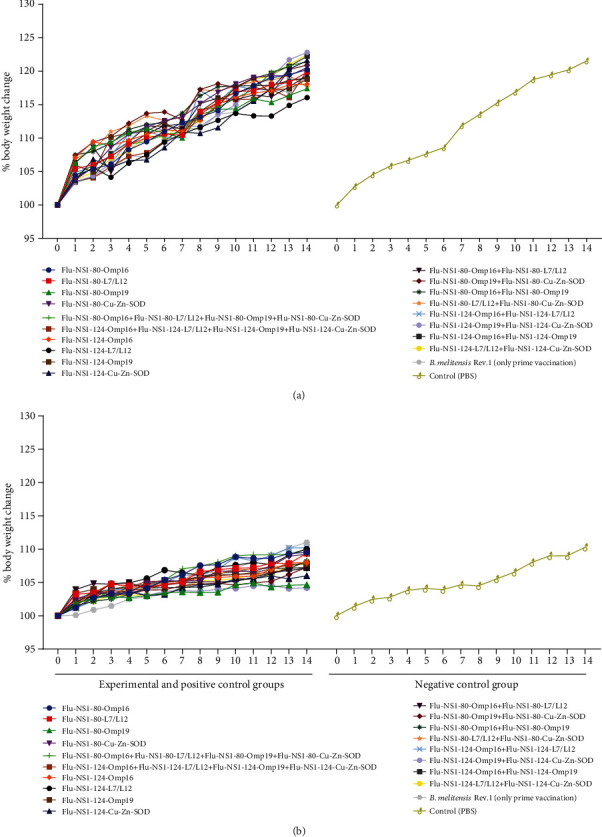
Percentages of body weight change of mice after prime-booster immunization. Percentages of body weight change of mice in experimental and control (after single vaccination with *B. melitensis* Rev.1 or administration of PBS) groups recorded daily 28 days after prime (a) and booster (b) immunization with mono-, bi-, or tetravalent formulations of rIVVs. Statistical analysis was performed with one-way ANOVA followed by Dunnett's multiple comparison test showed that during the 28 days, body weight measurement between the PBS control and vaccinated groups was not significant. *P* < 0.05 values were considered significant.

Assessment of percent change of body weight over 28 days upon prime or boost immunizations showed an increase in the animals' body weight for all type of viral construct (Figure 1). By the end of the observation period, the weight of mice in the experimental groups has been increased by 19-29% or 3.8-5.8 g, which was similar to the control group—28% or 4.7 g. No group was significantly different in mean of body weight from the PBS-treated control group (*P* > 0.5).

It should be noted that examination of the *Brucella* proteins in the NS1 gene by the RT-PCR confirmed that all the viral constructs retained their corresponding *Brucella* inserts upon producing rIVV in CE.

### 3.2. Protectiveness of Vaccine Formulations in Mice against *B. melitensis* 16M Infection

This experiment was conducted to assess protective efficacy of 18 mono-, bi-, or tetravalent vaccine formulations first in a mouse model to determine the most protective vaccine formulations against *B. melitensis* 16M infection and to compare to the reference *B. melitensis* Rev.1 vaccine group or PBS control group. The protective activity of the vaccines was evaluated by bacterial load or virulent *Brucella* bacteria colonization in spleens of vaccinated and nonvaccinated animals. The results of the bacteriology study after the virulence challenge showed that mono-, bi-, or tetravalent vaccine formulations as well as *B. melitensis* Rev.1 provided protection between 1.6 and 2.79 log_10_ units. Compared to the PBS unvaccinated control group, all vaccine formulations as well as the commercial vaccine provided protection of mice from *B. melitensis* 16M infection on rates of virulent strain colonization in challenged animal spleens (Table 2).

Significant protection in comparison with the PBS control group was achieved in the following 5 groups: (1) 80-Omp16, (8) 124-Cu-Zn-SOD, (13) 80-Omp16+L7/L12+Omp19+Cu-Zn-SOD, (16)124-Omp16+Omp19, and (18) 124-Omp16+L7/L12+Omp19+Cu-Zn-SOD (*P* < 0.02) as well as in the positive control group vaccinated with *B. melitensis* Rev.1 (*P* < 0.02). By the result of this study, the five most protective vaccine formulations that showed significant results in mice were continued in guinea pigs to confirm an efficacy of selected vaccine formulations.

### 3.3. Assessing of Protection of Five Vaccine Formulations in Guinea Pigs

**Figure 2:**
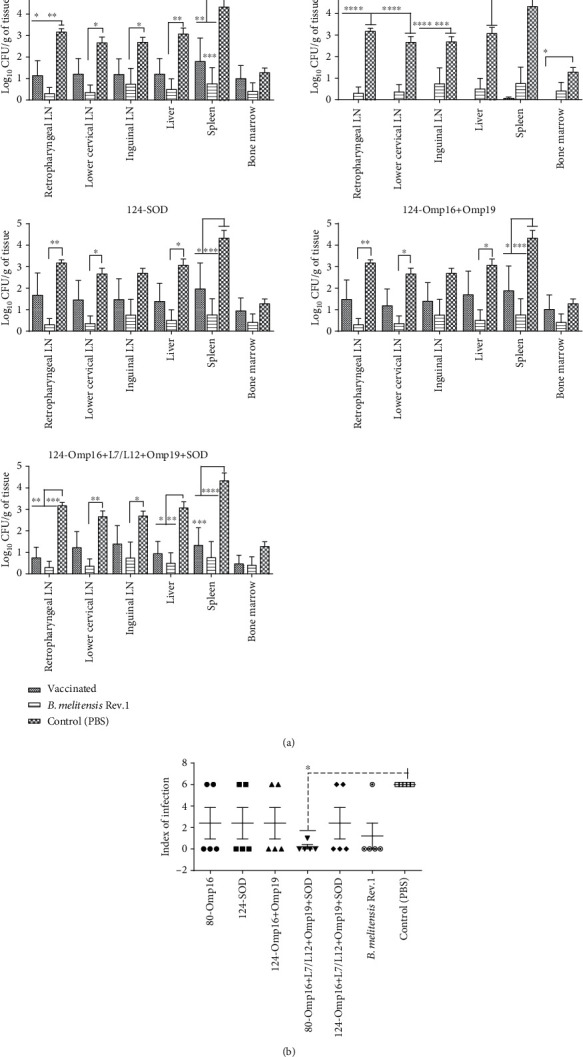
Protectiveness of vaccine samples in guinea pigs estimated by the amount allocated to *Brucella* from tissues and organs (a) and index of infection (b). Animals were vaccinated at regime of prime-boost at interval of 21 days with mono-, bi-, or tetravalent vaccine formulations or a single delivery of commercial vaccine *B. melitensis* Rev.1. Guinea pigs in the negative control group were delivered with PBS. The challenge of animals was performed with virulent strain of *B. melitensis* 16M at a dose of 1.3 log_10_ CFU/animal using an *s.c.* route. Bacteriological evaluation was assessed by counting *Brucella* colonies in tissues, where data is expressed as log_10_ CFU/g and the index of infection in animals (the arithmetic mean ± standarderror was given for each group; number of tissues from where *Brucella* was isolated for each animal). Statistical analysis for (a) was performed using a one-way ANOVA followed by Tukey's multiple comparison test, and for (b) two-way ANOVA followed by Sidak's multiple comparison test. From ^∗^*P* = 0.01 to *P* = 0.04; from ^∗∗^*P* = 0.002 to *P* = 0.004; from ^∗∗∗^*P* = 0.0002 to *P* = 0.0007; ^∗∗∗∗^*P* < 0.0001.

From the 18 vaccine formulations assessed in mice, the five most protective ones were further investigated in a guinea pig model. The protective efficacy of viral vectors expressing immunodominant *Brucella* proteins Omp16, L7/L12, Omp19, and SOD at mono-, bi-, or tetravalent vaccine formulations was compared to that of a commercial vaccine *B. melitensis* Rev.1 and PBS and assessed by parameters, such as colonization of the virulent strain of *B. melitensis* 16M in the lymph nodes and organs of the vaccinated and unvaccinated animals and index of infection (number of tissues and organs from which *Brucella* bacteria were isolated). All vaccine formulations and the commercial vaccine provided significant protection (*P* < 0.01–*P* < 0.0001 compared to the PBS control group) to the guinea pigs against the virulent strain *B. melitensis* 16M to a certain degree, specifically in the number of cultured *Brucella* in tissues and organs of animals upon the challenge. In animals vaccinated with 80-Omp16+L7/L12+Omp19+SOD (Figure 2(a), average value for the group: 0.01 log_10_ CFU/g of tissue) and 124-Omp16+L7/L12+Omp19+SOD (average value for group: 1.01 log_10_ CFU/g of tissue), we found a low degree of *Brucella* colonization in tissues (for the NS1-80 tetravalent group only in the spleen) in comparison with the PBS control group (2.9 log_10_ CFU/g of tissue).

According to the index of infection (Figure 2(b)), a significant level of protection in comparison with the control challenge group (the infection rate, 100%) was achieved in the group that was vaccinated with the tetravalent viral construct (*P* < 0.01; vaccination efficacy, 80%) expressing the *Brucella* Omp16, L7/L12, Omp19, and SOD proteins fused to the N-terminal 80 amino acids of NS1. It is worth noting that although in the animal group vaccinated with *B. melitensis* Rev.1, the effectiveness of vaccination (Table 3) reached 80% (*P* = 0.02); however, the index of infection for the *B. melitensis* Rev.1 group was not significantly different from that for the groups vaccinated with the above-mentioned tetravalent viral constructs.

## 4. Discussion

The available commercial vaccines against brucellosis are limited to use just in small ruminants and cattle because of the adverse effect of these vaccines for human use [30]. The search for safe and effective human brucellosis vaccines remains active today, specifically for farmers in endemic places as well as for veterinarians with risks related to the occupational exposure and animal care workers [31]. Numerous candidate vaccines and vaccine strategies against *B. abortus* have been evaluated in animal models, including DNA vaccine, recombinant subunit peptide, protein, LPS, outer membrane vesicles (OMV), a live vector (viral or bacterial vector-based *Brucella* vaccines), combinations in prime and boost strategies, and others. *Brucella* recombinant vaccinia viruses expressing L7/L12, Omp18, and GroEL proteins have been studied in mouse models without substantial protection against *Brucella* challenge [32–34]. Highly immunogenic constructs have been developed based on an adenoviral vector expressing both p39 and lumazine synthase proteins of *B. abortus* [35]. However, preexisting immunity to the viral vector could prevent a vaccine from working. To circumvent the problem of preexisting immunity, nonhuman adenovirus vectors or genetically modified adenovirus constructs could be used as the vaccine carrier. Replication-deficient *Semliki Forest* virus expressing *Brucella* translation initiation factor 3 (IF3) and Sod C generated some Th1 response and partial protection in mice [36, 37]. Influenza viruses expressing *Brucella* ribosomal proteins L7/L12 and Omp16 produced a long-term protection in pregnant heifers against *B. abortus* infection [38, 39]. As reported previously, immunization with *B. abortus* recombinant influenza A viruses based on subtypes H5N1 or H1N1 was protective in cattle [38]. However, bovines are naturally immune to influenza infections. We reasoned if we could generate protection in mouse and guinea pig models using improved *Brucella* antigen composition in influenza A viruses based on subtypes H5N1 as vaccine career, then we can potentially use these recombinant vaccines for preclinical and clinical trials in humans.

There is absence or little preexisting immunity against influenza virus A of subtype H5N1 in the human population. This is an important conceptual basis and quality of our vaccine vector candidates for future *Brucella* vaccine development in humans. We used a previously generated backlog of viral constructs, specifically 8 IVV of subtype H5N1 expressing Omp16, L7/L12, Omp19, and Cu-Zn SOD inserted into the NS1 gene region at position 80 or 124 amino acids. From these viral constructs were formed 18 vaccine formulations to characterize their safety and protection in mice and guinea pigs to form the final vaccine formulation. As the vaccine intended for use in humans, placing outbred animals (BALB/c mice) and a less homogeneous population of guinea pigs should demonstrate relationship in consistency and performance.

In the present study, we used intraperitoneal (*i.p.*) inoculation of rIVV in the prime-boost immunization strategy in mice and intranasal (*i.n.*) administration in the guinea pig model. Animals in the control group have been vaccinated with commercial *B. melitensis* Rev.1 *s.c.* by the manufacturer's recommendations. For this study, the influenza A viral vector was generated on the backbone of the NS (chimeric) gene of Puerto Rico/8/34 (H1N1), and the surface genes for hemagglutinin and neuraminidase were taken from A/chicken/Astana/6/05 (H5N1) strain. The PR8 virus is a mouse-adapted virus with efficient replicative properties in mice that could cause an infectious process in mouse lungs and lead to mortality when delivered *i.n.* [40–43]. Therefore, we used *i.p.* inoculation of vaccine samples in mice due to the nature of the PR8 virus to cause disease symptoms.

As the influenza virus has tropism to mucosal surfaces, we believe that the optimal way for rIVV immunization is the *i.n.* route. Bearing in mind that *Brucella* should be considered as a mucosal pathogen, penetrating the mucosa of the nasal or oral cavities after ingestion, mucosal vaccination is capable of generating protective responses against pathogens at the mucosal site of entry [44].

Safety or attenuation of rIVV conferred by the shortened NS1 gene that facilitates their limited replicative ability [29]. We found that all vaccine formulations were safe, and no animal death or weight decrease was observed in mice. Additionally, after the challenge study in mice, we took five effective vaccine formulations and used them for *i.n.* immunization of guinea pigs. Importantly, bacteriological studies in mice demonstrated that that rIVV-based vaccine formulations demonstrated a similar level of protection as commercially established vaccine *B. melitensis* Rev.1. These data were reproducible in the guinea pig model as well. Thus, we concluded that rIVV vaccine formulations were as effective as the commercial vaccine. Formulations coding amino acids at positions 80 or 124, especially tetravalent constructs, in protection studies demonstrated results similar to those of the group vaccinated with the commercial vaccine. In addition, protective efficacy and immunogenicity of the candidate vaccine were established through a standardized challenge study by the ability of an animal model to restrain bacteria in the spleen [45].

To our knowledge, this is the first study to utilize a *Brucella* recombinant vector coding 80 amino acids at ORF of the NS1 for protective studies in animal models. For development of a human vaccine, NS1-80 may have some advantages. Knowing interferon antagonist properties of the influenza NS1 protein, viral constructs with a length of NS1-80 amino acid size should be less aggressive than NS-124, which has a half-length of the NS1. There is some correlation between length of the NS1 protein and attenuation of the virus in live organisms [46]. Because IVV has a different infectivity based on the length of the NS1 protein, combined use of IVV with different lengths of the NS1 protein (80 and 124) can lead to the interference of individual viruses, which is not observed when using IVV with the same length of NS1 protein.

There are some concerns in general about using IVV of subtype H5N1, which is a pathogenic type of influenza virus circulated in birds. Major concerns related to interspecies transmission of the disease from bird to human may cause diseases in the human population. However, in our case, the virus has proven to be attenuated through removal of the proteolytic cleavage site in the HA, and safety of the rIVV is ensured through the shortened NS1 gene, and as the result, they have limiting replicative ability [29]. Another risk related to using rIVV to public health is generation of reassortment between avian H5N1 and human influenza viruses that might confer pandemic strains [47]. However, for more than 30 years of using live attenuated cold-adapted influenza virus vaccine for humans, there are no records to date of a new emergence of virulent reassortants [48]. This vaccine candidate is not intended for mass vaccination and will be used for people in the risk groups only. Our previous studies have demonstrated that after repeated passages in CE, the IVV retained its main biological properties, including attenuation, and did not lose *Brucella* antigen inserts [29], indicating their genetic stability. In addition, the IVV belong to the group of RNA viruses, which facilitates the limiting replicative process that eliminates the risk of integration and long-term persistence.

## 5. Conclusions

Thus, the results of this study demonstrated that recombinant influenza virus subtype A/H5N1 expressing the *Brucella* L7/L12 or Omp16 or Omp19 or Cu-Zn SOD (SOD) proteins from the open reading frame (ORF) of the NS1 gene in combination with tetravalent formulations is a safe vector, and its protectiveness against *B. melitensis* 16М infection in the prime-boost regimen is comparable to the *B. melitensis* Rev.1 commercial vaccine in mouse and guinea pig models. This study is a substantial step for the development of a safe and protective human brucellosis vaccine.

## Figures and Tables

**Table 1:** Scheme of immunization of animals.

Species	Viral construct	Prime vaccination dose (log_10_ EID_50_/animal)	Booster vaccination dose (log_10_ EID_50_/animal)
Mice *i.p.*	Monovalent vaccine formulation (VF)		
	(1) Flu-NS1-80-Omp16	(1) 6.14	(1) 6.22
	(2) Flu-NS1-80-L7/L12	(2) 6.06	(2) 6.14
	(3) Flu-NS1-80-Omp19	(3) 6.64	(3) 6.56
	(4) Flu-NS1-80-SOD	(4) 6.56	(4) 6.22
	(5) Flu-NS1-124-Omp16	(5) 6.64	(5) 6.56
	(6) Flu-NS1-124-L7/L12	(6) 6.69	(6) 6.64
	(7) Flu-NS1-124-Omp19	(7) 6.22	(7) 6.31
	(8) Flu-NS1-124-SOD	(8) 6.31	(8) 6.56

Mice *i.p.*	Bivalent VF		
	(9) Flu-NS1-80-Omp16+Flu-NS1-80-L7/L12	(9) 5.84 + 5.76	(9) 5.92 + 5.84
	(10) Flu-NS1-80-Omp19+Flu-NS1-80-SOD	(10) 6.34 + 6.26	(10) 6.26 + 5.92
	(11) Flu-NS1-80-Omp16+Flu-NS1-80-Omp19	(11) 5.84 + 6.34	(11) 5.92 + 6.26
	(12) Flu-NS1-80-L7/L12+Flu-NS1-80-SOD	(12) 5.76 + 6.26	(12) 5.84 + 5.92
	(13) Flu-NS1-124-Omp16+Flu-NS1-124-L7/L12	(13) 6.34 + 6.39	(13) 6.26 + 6.34
	(14) Flu-NS1-124-Omp19+Flu-NS1-124-SOD	(14) 5.92 + 6.01	(14) 6.01 + 6.26
	(15) Flu-NS1-124-Omp16+Flu-NS1-124-Omp19	(15) 6.34 + 5.92	(15) 6.26 + 6.01
	(16) Flu-NS1-124-L7/L12+Flu-NS1-124-SOD	(16) 6.39 + 6.01	(16) 6.34 + 6.26

Mice *i.p.*	Tetravalent VF		
	(17) Flu-NS1-80-Omp16+Flu-NS1-80-L7/L12+Flu-NS1-80-Omp19+Flu-NS1-80-SOD	(17) 5.54 + 5.46 + 6.04 + 5.96	(17) 5.62 + 5.54 + 5.96 + 5.62
	(18) Flu-NS1-124-Omp16+Flu-NS1-124-L7/L12+Flu-NS1-124-Omp19+Flu-NS1-124-SOD	(18) 6.04 + 6.09 + 5.62 + 5.71	(18) 5.96 + 6.04 + 5.71 + 5.96

Guinea pigs^∗^ *i.n.*	(1) Flu-NS1-80-Omp16	(1) 6.75	(1) 6.83
	(8) Flu-NS1-124-SOD	(8) 6.92	(8) 7.04
	(15) Flu-NS1-124-Omp16+Flu-NS1-124-Omp19	(15) 6.95 + 6.53	(15) 6.87 + 6.62
	(17) Flu-NS1-80-Omp16+Flu-NS1-80-L7/L12+Flu-NS1-80-Omp19+Flu-NS1-80-SOD	(17) 6.14 + 6.06 + 6.64 + 6.56	(17) 6.22 + 6.14 + 6.56 + 6.22
	(18) Flu-NS1-124-Omp16+Flu-NS1-124-L7/L12+Flu-NS1-124-Omp19+Flu-NS1-124-SOD	(18) 6.64 + 6.69 + 6.22 + 6.31	(18) 6.56 + 6.64 + 6.31 + 6.56

Table note: number of animals per viral construct vaccine formulation for mice was 5 or 12 and for guinea pigs was 5 per group. The amounts of substance for mice via intraperitoneal (*i.p.*) were 500 *μ*l, and for guinea pigs, intranasal (*i.n*.) immunization was 200 *μ*l in both nostrils. ^∗^After the evaluation of protective efficacy of 18 vaccine formulations in mice, the 5 most protective vaccine formulations were selected and then tested in guinea pigs.

**Table 2:** Level of protective efficacy of vaccines assessed by the isolation rate of *Brucella* from the spleens of mice challenged with the virulent strain *B. melitensis* 16M.

Groups	Vaccine samples	No. of animals	Brucella titer, log_10_ CFU/g spleen (mean ± SE)	Protection unit (log_10_)^∗^	Significance to control group	
					(+) control	(-) control
1	80-Omp16	5	3.08 ± 0.86	2.79^∗^	>0.05	<0.05
2	80-L7/L12	5	3.7 ± 0.33	2.17	>0.05	>0.05
3	80-Omp19	5	3.68 ± 0.37	2.2	>0.05	>0.05
4	80-SOD	5	3.64 ± 0.38	2.24	>0.05	>0.05
5	124-Omp16	5	3.41 ± 0.84	2.46	>0.05	>0.05
6	124-L7/L12	5	3.47 ± 0.55	2.40	>0.05	>0.05
7	124-Omp19	5	4.1 ± 0.29	1.78	>0.05	>0.05
8	124-SOD	5	3.2 ± 0.76	2.60^∗^	>0.05	<0.05
9	80-Omp16+L7/L12	5	3.78 ± 0.41	2.1	>0.05	>0.05
10	80-Omp19+SOD	5	4.18 ± 0.3	1.7	>0.05	>0.05
11	80-Omp16+Omp19	5	3.83 ± 0.39	2.04	>0.05	>0.05
12	80-L7/L12+SOD	5	3.57 ± 0.28	2.30	>0.05	>0.05
13	80-Omp16+L7/L12+Omp19+SOD	5	3.13 ± 0.25	2.75^∗^	>0.05	<0.05
14	124-Omp16+L7/L12	5	3.49 ± 0.36	2.38	>0.05	>0.05
15	124-Omp19+SOD	5	3.64 ± 0.53	2.23	>0.05	>0.05
16	124-Omp16+Omp19	5	3.18 ± 0.69	2.69^∗^	>0.05	<0.05
17	124-L7/L12+SOD	5	4.27 ± 0.3	1.60	>0.05	>0.05
18	124-Omp16+L7/L12+Omp19+SOD	5	3.17 ± 0.38	2.70^∗^	>0.05	<0.05
19	*B. melitensis* Rev.1	5	3.20 ± 0.31	2.68^∗^	—	<0.05
20	Control (PBS)	5	5.88 ± 0.16	—	<0.05	—

Table legend: ^∗^log_10_ protection units were obtained by subtracting the mean log_10_ CFU of the control (PBS) group from the mean of log_10_ CFU for the experimental group and for the positive control group. (+) control: animals vaccinated with *B. melitensis* Rev.1 commercial vaccine. (-) control: animals inoculated with PBS. ^∗^alpha = 0.02‐0.01 vs. PBS control group, *B. melitensis* Rev.1 vs. vaccine groups. Statistical analysis was performed using a one-way ANOVA (Tukey's multiple comparison test).

**Table 3:** Rates of protection in guinea pigs after challenge with the virulent strain *B. melitensis* 16M.

Immunization group	Total animals	Isolation of *B. melitensis* in animals, *n* (%)	Value (*P*)^∗^	
			(+) control	(-) control
80-Omp16	5	2 (40)	>0.05	>0.05
80-Omp16+L7/L12+Omp19+SOD	5	1 (20)	>0.05	<0.05
124-SOD	5	2 (40)	>0.05	>0.05
124-Omp16+Omp19	5	2 (40)	>0.05	>0.05
124-Omp16+L7/L12+Omp19+SOD	5	2 (40)	>0.05	>0.05
*B. melitensis* Rev.1	5	1 (20)	—	<0.05
Control (PBS)	5	5(100)	<0.05	—

Table note: ^∗^in comparison with control untreated PBS or *B. melitensis* Rev.1 groups. Statistical analysis was performed using a one-sided Fisher's exact test. <0.05: *P* value less than 0.05; >0.05: *P* value higher than 0.05.

## Data Availability

All datasets generated for this study are included in the article/supplementary material.

## Supplementary Materials

Supplementary Materials Figure S1: insertion of Brucella proteins in the NS1 gene as determined by RT-PCR upon accumulation in CE.
