# Supplementary material for: Development of Human Vectored Brucellosis Vaccine Formulation: Assessment of Safety and Protectiveness of Influenza Viral Vectors Expressing Brucella Immunodominant Proteins in Mice and Guinea Pigs
Source: Biomed Res Int. 2020 Nov 19;2020:1438928. doi: 10.1155/2020/1438928 (PMC7695499; doi:10.1155/2020/1438928)
Supplement: Supplementary Materials — Figure S1: insertion of Brucella proteins in the NS1 gene as determined by RT-PCR upon accumulation in CE. [file 1438928.f1.pptx]

## Slide 1
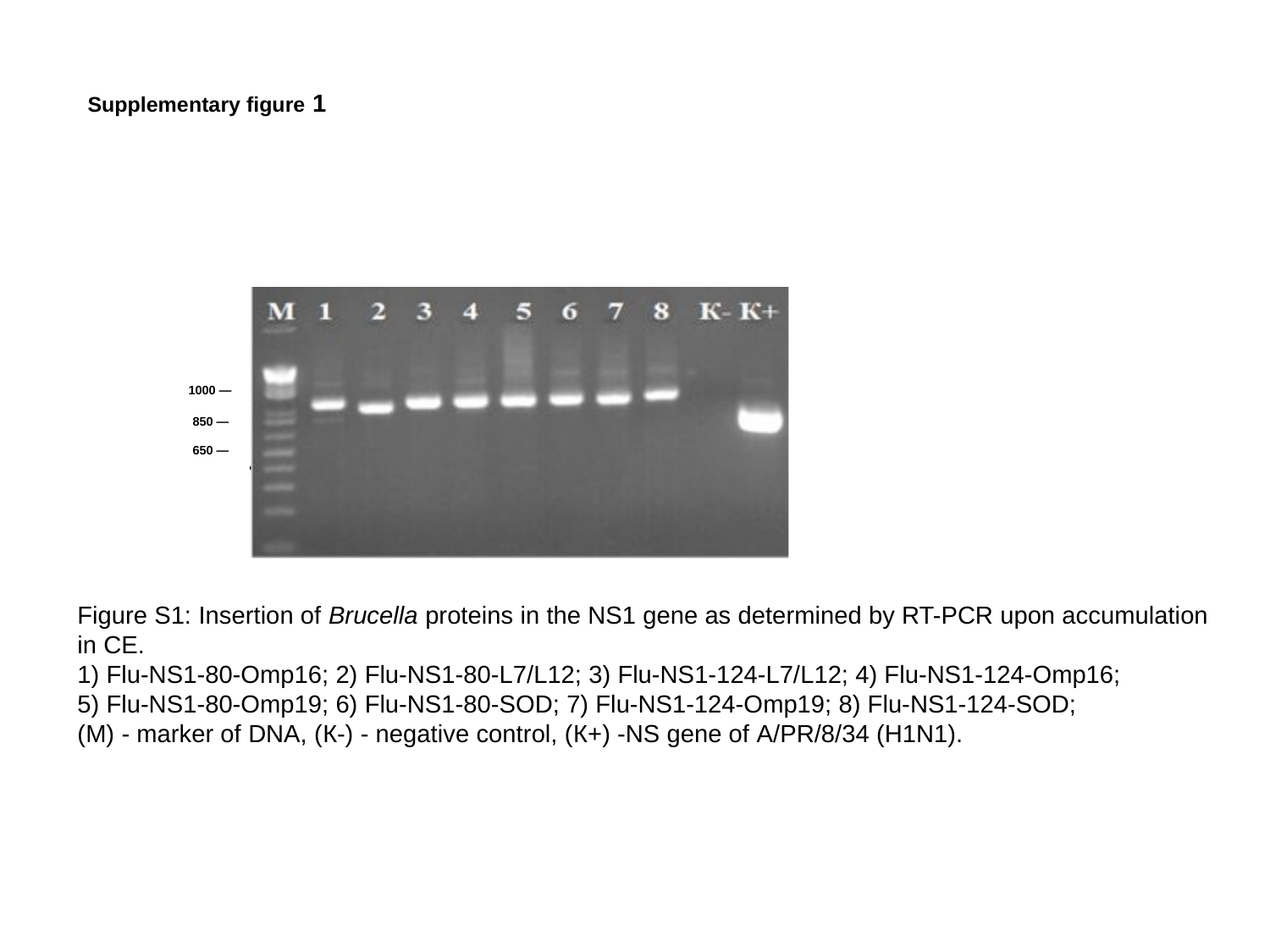

Supplementary figure 1
 1000 ­­—
 850 —
 650 —
Figure S1: Insertion of Brucella proteins in the NS1 gene as determined by RT-PCR upon accumulation in CE.
1) Flu-NS1-80-Omp16; 2) Flu-NS1-80-L7/L12; 3) Flu-NS1-124-L7/L12; 4) Flu-NS1-124-Omp16; 5) Flu-NS1-80-Omp19; 6) Flu-NS1-80-SOD; 7) Flu-NS1-124-Omp19; 8) Flu-NS1-124-SOD; (M) - marker of DNA, (К-) - negative control, (К+) -NS gene of А/РR/8/34 (H1N1).
